# Striving for vessel health preservation through standardized assessment: a Letter to the Editor

**DOI:** 10.1590/0034-7167.2023760301c

**Published:** 2023-06-21

**Authors:** Paulo Jorge dos Santos-Costa, Margarida Maria da Silva Vieira, João Manuel Garcia Nascimento Graveto

**Affiliations:** I Nursing School of Coimbra. Coimbra, Portugal; II Universidade Católica Portuguesa. Porto, Portugal

Dear Dr Dulce Aparecida Barbosa

Editor in Chief of the Revista Brasileira de Enfermagem

It was with great interest that we read the article “Validation of the Brazilian Portuguese version of the Venous International Assessment Scale and proposal of revision”, published by Lopes and colleagues in the latest issue of the journal.

First, we applaud the authors on the work conducted. Peripheral intravenous catheterization (PIVC) is considered the most recurrently performed invasive procedure in hospital settings, supporting numerous therapeutic and diagnostic techniques. Still, one in every four patients is subjected to consecutive puncture attempts by clinicians, leading to vessel health harm and depletion over time, with significant impact on patients (e.g., pain, distrust, delays in treatment), healthcare professionals (e.g., anxiety, increased care volume and complexity) and health organizations (e.g., longer admission periods, increased care costs)^(1-2)^. Thus, implementing formal assessment of venous assessment with standardized instruments will likely increase the identification of patients at higher risk of difficult intravenous access (DIVA) and the adoption of differentiated techniques (e.g., ultrasound-guided PIVC), leading to better patient outcomes and care experience^(1,3)^.

After careful analysis of Lopes and colleagues’ work, we conclude that the proposed VIA-R scale is yet to be implemented and tested in Brazilian healthcare settings, contrary to what has been done in previous studies^(4-5)^. Besides the satisfactory findings reported by the authors on the VIA-R’s content validity, we believe that the authors should focus on the scale’s reliability (e.g., predictive, concurrent, convergent, discriminant) before considering it a “validated instrument” (p.7) that can be used by certified healthcare professionals in Brazil. This open letter allows Lopes and colleagues to present their plans for the assessment of the VIA-R’s reliability since such considerations are omitted and offer nurses and other healthcare professionals in Brazil insights on how the scale should be implemented in clinical practice.

We respectfully make ourselves available for any necessary clarifications and look forward to Lopes and colleagues’ future work.
