# Supplementary material for: Striving for vessel health preservation through standardized assessment: a Letter to the Editor
Source: Rev Bras Enferm. 2023 Jun 21;76(3):e2023760301c. doi: 10.1590/0034-7167.2023760301c (PMC10292614; doi:10.1590/0034-7167.2023760301c)
Supplement: Related texts

# Reply: Validation of the Brazilian Portuguese version of the Venous International Assessment Scale and proposal of revision

**Authors:** Mayara Lopes, Julio César de la Torre-Montero, Maria Angélica Sorgini Peterlini, Mavilde da Luz Gonçalves Pedreira

**Affiliations:** I Universidade Federal de São Paulo. São Paulo, São Paulo, Brazil; II Comillas Pontifical University. Madrid, Spain; III Conselho Nacional de Desenvolvimento Científico e Tecnológico CNPq. São Paulo, São Paulo, Brazil

Dear Dr Dulce Aparecida Barbosa

Editor in Chief of the Revista Brasileira de Enfermagem

We are grateful for your interest in reading our article about the venous assessment for peripheral intravenous puncture and for the opportunity to clarify some achievements in our research, in addition to sharing future plans regarding this study.

Investigating about the various aspects that involves peripheral intravenous puncture and how it is relevant for the health area, both in terms of clinical practice and the patient’s experience, identifying scientific evidence that made intervention practices fundamental and achieving better quality and safety indicators, since this is estimated to be one of the most frequent procedures in health care, with more than 300 million peripheral intravenous punctures performed by health professionals yearly^(1)^.

It should be considered as a highlight that the decision to choose the catheterization site and the infusion mode of solutions and medications are factors that can contribute to better results during intravenous therapy, by allowing the risk factors controls for complications, such as infiltration and phlebitis, preventing the occurrence of adverse events that compromise patient safety^(2)^.

Obtaining the evidence that promotes better results from the use of peripheral intravenous catheters, with insertion in the first puncture attempt and maintenance until the end of the therapeutic needs, preventing adverse events and promoting vascular health, are the assumptions that underlie research in this area pressing for the clinical and technological advances.

Furthermore, a study that demonstrated the insertion and maintenance processes of peripheral intravenous catheters in 132 hospitals in five Latin American countries (Argentina, Brazil, Chile, Colombia and Mexico), covering 9620 peripheral intravenous catheters, identified results indicating the need for advances in evidence-based clinical practice to promote better indicators, since there are more than 25% failure in the first puncture, 29% of the catheters are inserted in the veins of the hand, 19% stabilized with non-sterile tapes, 18% of the observed catheters had signs of adverse events, reaching a an average length of stay of one day^(3)^.

The main objective of our study was to validate the Venous International Assessment (VIA) scale for the Brazilian Portuguese. During the study, we noticed the need to make changes to adapt it culturally and in consensus with the original author’s scale, we proposed a revised scale.

Thus, we agree with the perspective pointed out that we didn’t obtain a validated instrument, rather, we achieved the content validation of the scale for Brazilian Portuguese, requiring that the proposed version to be observed with additional tests that make it possible to support the implementation in clinical practice of Brazilian health institutions.

The method of study followed the recommendations of Beaton and Guillemin^(4)^ and the statistical analysis applied to the agreement between evaluators regarding the scale content, allows us to infer that the validation of the cultural adaptation was completed^(5)^.

In order to ensure the use of the scale in clinical practice, it is required that it be, in fact, applied with patients and carried out with observation of possible results, allowing us to evaluate other important psychometric properties of the instrument, such as reliability^(6)^, obtaining scientific data that demonstrate the real contribution of the VIA-R scale in directing conducts and interventions that promote vascular health throughout the therapy performed through peripheral intravenous catheters, effectively the results of professional practice and the patient’s experience with the care provided.

We emphasize that these results are promising, since in addition to the original study, the VIA Scale was validated for European Portuguese, called EARV, with clinical application in 100 patients, demonstrating statistically significant results such as success in inserting the peripheral intravenous catheter in one or two attempts (an average of 1.57) and a length of stay 2.1 days^(7)^.

We plan to carry out these next validation processes initiated in the main author’s master’s thesis as broader research to be implemented in her doctoral thesis, and we hope to be able to count on analysis such this and future contributions to the advancement of science and practice in intravenous therapy.

## References

[1] Alexandrou E, Ray-Barruel G, Carr PJ, Frost SA, Inwood S, Higgins N (2018). Use of short peripheral intravenous catheters: characteristics, management, and outcomes worldwide. J Hosp Med.

[2] Simões AMN, Vendramim P, Pedreira MLG (2022). Risk factors for peripheral intravenous catheter-related phlebitis in adult patients. Rev Esc Enferm USP.

[3] Walker RM, Pires MPO, Ray-Barruel G, Cooke M, Mihala G, Azevedo SS (2023). Peripheral vascular catheter use in Latin America (the vascular study): a multinational cross-sectional study. Front Med.

[4] Beaton DE, Bombardier C, Guillemin F, Ferraz MB (2000). Guidelines for the process of cross-cultural adaptation of self-report measures. Spine [Internet].

[5] Yusoff MSB (2019). ABC of Content Validation and Content Validity Index Calculation. EIMJ.

[6] Souza AC, Alexandre NMC, Guirardello EB (2017). Psychometric properties in instruments evaluation of reliability and validity. Propriedades psicométricas na avaliação de instrumentos: avaliação da confiabilidade e da validade. Epidemiol Serv Saude.

[7] Santos-Costa P, Sousa LB, Torre-Montero JC, Salgueiro-Oliveira A, Parreira P, Vieira M (2021). Translation, cultural adaptation, and validation of the Venous International Assessment Scale to European Portuguese. Rev Enferm Referência.
